# Seasonal Change in Home Blood Pressure Monitoring Is Associated With Renal Outcome and Mortality in Patients With Chronic Kidney Disease

**DOI:** 10.3389/fmed.2021.672651

**Published:** 2021-05-28

**Authors:** Chun Yin See, Chien-Tzu Tseng, Wei-Ren Lin, Jo-Yen Chao, Te-Hui Kuo, Ming-Cheng Wang

**Affiliations:** ^1^Division of Nephrology, Department of Internal Medicine, National Cheng Kung University Hospital, College of Medicine, National Cheng Kung University, Tainan, Taiwan; ^2^Department of Public Health, College of Medicine, National Cheng Kung University, Tainan, Taiwan; ^3^Institute of Clinical Pharmacy and Pharmaceutical Sciences, College of Medicine, National Cheng Kung University, Tainan, Taiwan

**Keywords:** hypertension, seasonal blood pressure variations, home blood pressure monitoring, chronic kidney disease, outcome

## Abstract

**Background:** Blood pressure (BP) variation may result in poor cardiovascular and renal outcomes. We investigated the pattern of seasonal BP change and its association with outcomes in patients with chronic kidney disease (CKD) living in southern Taiwan.

**Methods:** We conducted a retrospective analysis of a prospective observational cohort consisting of outpatients with CKD for the period between December 2014 and December 2019. These patients were grouped according to the pattern of seasonal BP variation, namely, consistently higher average systolic BP (≥8 mmHg) in wintertime than summertime (Group A), consistently lower average systolic BP (≥8 mmHg) in wintertime than summertime (Group B), large variation of average systolic BP (≥8 mmHg) without a specific pattern related to weather (Group C), and little fluctuation of average systolic BP (<8 mmHg) throughout the years (Group D). The study endpoints were ≥40% reduction in estimated glomerular filtration rate (eGFR), end stage renal disease (initiation of dialysis or transplantation), or death.

**Results:** We analyzed 507 eligible patients, of whom 17.2% exhibited consistent BP elevation in the wintertime. There were 56.8% of patients conducting regular home BP monitoring. Cox regression analysis showed home BP monitoring was independently associated with better outcome in 507 CKD patients (HR 0.72, 95% CI 0.56–0.94, *P* = 0.0162). Compared with the other three groups, patients with BP elevation in the wintertime (Group A) were older, had a higher prevalence of diabetic nephropathy and nephrotoxic agent use, a lower prevalence of statin use, higher eGFR decline rate, and a worse outcome. Patients with BP reduction in the wintertime (Group B) were associated with the best outcome. Cox regression analysis indicated that consistent BP elevation in the wintertime in 288 CKD patients with home BP monitoring was significantly associated with a worse composite outcome (i.e., ≥40% reduction in eGFR, end stage renal disease, or death) after adjustment for various confounding factors.

**Conclusion:** Home BP monitoring is crucial, and associated with better outcome in CKD patients. Consistent home BP elevation from summertime to wintertime in patients with CKD was associated with a poorer composite outcome.

## Introduction

Blood pressure (BP) is a dynamic physical parameter that fluctuates according to environmental changes as well as the physical and emotional status of an individual. Greater BP variation may result in poorer cardiovascular and renal outcomes (1, 2). Self-monitoring of BP at home is an essential monitoring option, especially for patients with suspected white coat or masked hypertension. Home BP monitoring provides more accurate predictors of cardiovascular risk than does office BP measurement (3, 4). Seasonal changes also influence BP: higher BP is typically observed during cold weather. This effect is prominent even in normotensive individuals in countries with continental or temperate climates (3, 5). Nevertheless, higher BP level in response to cold weather is not a universal phenomenon. An inverse pattern of BP change with cold temperature (i.e., lower BP during wintertime) may be observed in some people (6).

The mechanism of BP elevation in winter may be related to sympathetic activation and release of hormones such as arginine vasopressin, norepinephrine, epinephrine, and angiotensin II in response to cold weather (7). Cold exposure might also cause endothelial dysfunction and perpetuate high BP (8). During cold seasons, people have lower vitamin D levels because of inadequate skin exposure to ultraviolet B radiation (7). Vitamin D is a potent endocrine suppressor of renin biosynthesis in the regulation of the renin–angiotensin system (RAS). RAS dysregulation causes sympathetic overactivity and endothelial dysfunction, leading to vasoconstriction and hypertension (9, 10).

Tainan, located in southern Taiwan, has a tropical monsoon climate, where extreme weather, such as heat waves or blizzards, is unlikely to occur. Despite moderate temperature variations, a lower ambient temperature in the wintertime still results in considerable BP elevation, as was demonstrated in a Taiwanese observational study of patients with chronic cardiovascular diseases (11). In the current study, we investigated the pattern of seasonal BP change and its association with outcomes in patients with chronic kidney disease (CKD) living in southern Taiwan.

## Materials and Methods

### Study Subjects

This study, involving a retrospective analysis of a prospective observational cohort, was conducted at National Cheng Kung University Hospital. It was reviewed and approved by the Institutional Review Board of National Cheng Kung University Hospital, Tainan, Taiwan (B-ER-109-402). The study population was patients with CKD aged ≥20, generally with CKD stage ≥3b. These patients had been enrolled in the Taiwan pre-end-stage renal disease management program and received regular follow-up at the nephrology outpatient clinic. The program was staffed by a multidisciplinary team, including nephrologists, renal nurses, and dieticians, who cared for patients with various CKD stages (11). We excluded patients who started dialysis therapy, underwent kidney transplantation, died, or were lost to follow up within the first 2 years of the study period. The patients' demographic and clinical data for the period from December 2014 to December 2019 were extracted from their electronic medical records.

### Clinical Parameters

We collected data on age, sex, comorbidity, cause of renal failure, medication use, and exposure to nephrotoxins or radiocontrast agents. BP measurement was performed during every outpatient visit using a standard sphygmomanometer device and measurement technique. Patients received relevant guidance and were instructed to measure and record their home BP once a week at least. Home BP was based on an average of ≥2 measurements obtained on different time points. For each monitoring period (wintertime and summertime), the arithmetic mean of all home BP readings was calculated (12). Then the average of home BP in wintertime and summertime during the first 2 years of enrollment were adopted as the values of home BP in wintertime and summertime, respectively. The frequency of blood sampling depended on CKD stage. Generally, patients with stage 3b, 4, and 5 CKD were followed up every 12 weeks, 8 weeks, and 4 weeks, respectively, or when necessary. Blood tests were performed every 12 weeks or when necessary.

### Definitions

CKD was defined according to the guideline (13). CKD stage was classified based on the estimated glomerular filtration rate (eGFR) from stage 1 to stage 5 (stage 1 with eGFR more than 90 ml/min, stage 2 with eGFR of 60 to 89 ml/min, stage 3 with eGFR of 45 to 59, stage 4 with eGFR of 15 to 29, and stage 5 with eGFR <15 ml/min) (13). The eGFR was calculated using the formula derived from the Modification of Diet in Renal Disease Study below:

$$\begin{array}{l} \text{eGFR}=186\times {\left(\text{serum}\;\text{creatinine}\right)}^{-1.154}\times {\left(\text{age}\right)}^{-0.203}\times 0.742\\ \;(\text{if}\;\text{female}) \end{array}$$

Wintertime was defined as the period from December 1 to February 28 (29), and summertime as the period from June 1 to September 30 on the basis of the average ambient temperature in southern Taiwan as published by the Central Weather Bureau of Taiwan. Home BP was adopted for patient classification and analysis, but office BP was applied in patients with CKD who did not perform home BP monitoring. The patients were divided into four groups according to the pattern of seasonal BP variation within the first 2 years of enrollment. BP elevation in the wintertime was defined as a consistently elevated wintertime (≥8 mmHg) average systolic BP compared with that in the summertime (group A). BP reduction in the wintertime was defined as a consistently lower (≥8 mmHg) average systolic BP in the wintertime (group B). Large BP variation was defined as a change in average systolic BP of 8 mmHg or more from summertime to wintertime without a specific pattern related to weather (group C). Stable BP was defined as a change in average systolic BP of <8 mmHg from summertime to wintertime throughout the years (group D). Except the group C, a consistent pattern of seasonal BP change was required for the groups A, B, and D. After the first 2 years of enrollment, the patients were followed up until the end of the study period or when they met any of the following endpoints, which were regarded as poor outcomes. A composite outcome was defined as: ≥40% reduction in eGFR, end stage renal disease (initiation of dialysis or transplantation) or death, whichever came first.

### Statistical Analysis

Data are presented as mean ± standard deviation, median (interquartile range), or number (percentage) when appropriate. Pearson's chi-square test or Fisher's exact test (two-tailed) was used for categorical variables. Student's *t*-test or one-way analysis of variance was conducted for normally distributed data, and the non-parametric Mann–Whitney *U*-test or Kruskall–Wallis test was used for continuous variables. We used Cox regression analysis (on the stratum of BP elevation in the wintertime) for factors associated with poor outcome (including age, gender, smoking, and underlying comorbidities/conditions that could affect the outcomes) in all the included patients and Cox proportional hazard model by adjusting for various subsets of covariates to analyze the association between BP elevation in the wintertime and outcomes in CKD patients with home BP monitoring. A *p*-value of <0.05 indicated statistical significance. All statistical analyses were performed using JMP (SAS institute, Cary, NC, USA).

## Results

**Figure 1:**
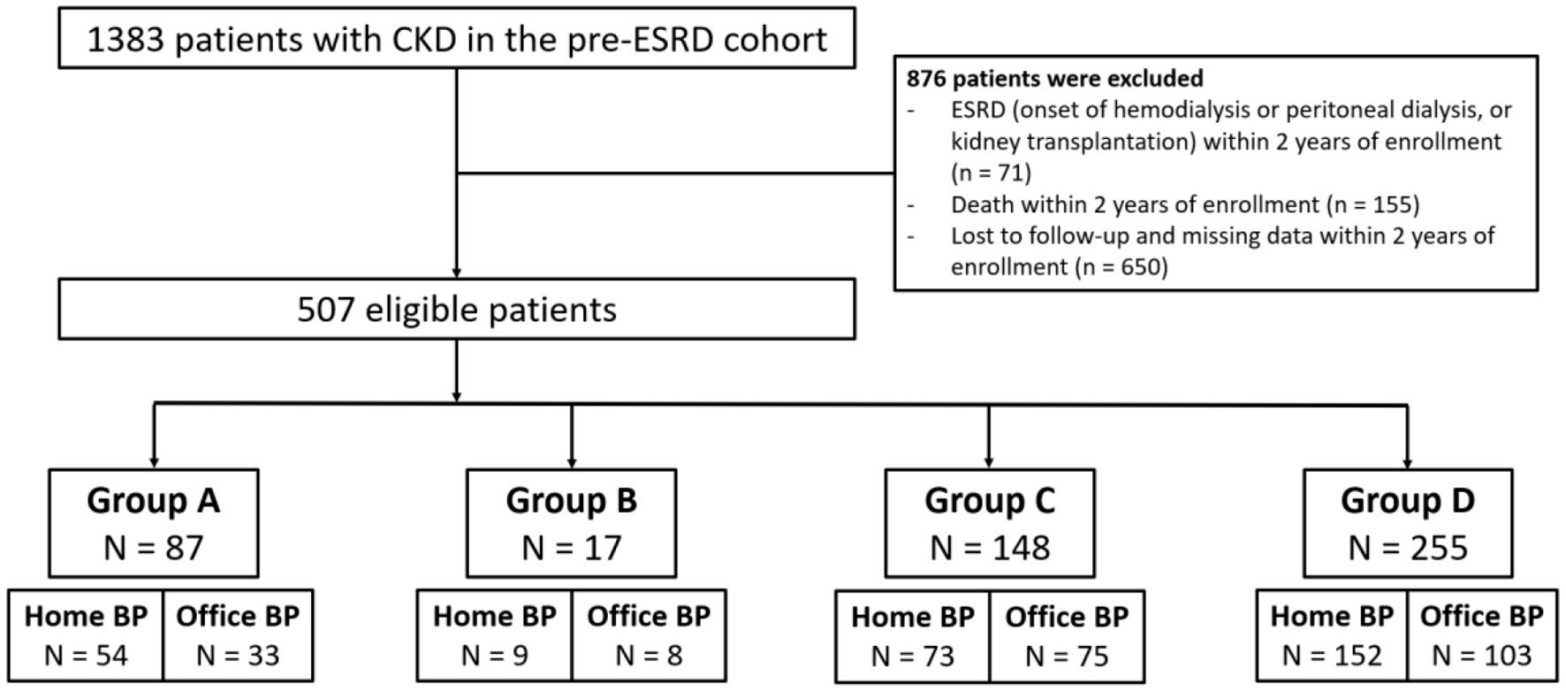
Flow chart for patient selection. CKD, chronic kidney disease; ESRD, end stage renal disease; BP, blood pressure; group A, BP elevation in the wintertime; group B, BP reduction in the wintertime; group C, large variation of BP; group D, stable BP.

We enrolled 1,383 patients with CKD who underwent regular follow-up at our nephrology clinic between December 1, 2014, and December 31, 2019. We excluded 876 patients because of missing data, initiation of dialysis, kidney transplantation, death, or loss to follow up within the first 2 years of enrollment. The remaining 507 patients were enrolled, and their baseline characteristics are listed in Table 1. We categorized these patients into four groups according to the pattern of seasonal BP change (Figure 1). Only 17.2% of our patients exhibited consistent BP elevation in winter (group A), and 3.4% of patients had lower BP in the wintertime (group B). Most of the patients (50.3%) had stable BP throughout the year (group D), and 29.2% of patients had a large BP variation without a specific pattern related to weather (group C).

**Table 1:** Baseline characteristics of 507 CKD patients in relation to the pattern of seasonal BP change.

**Characteristic**	**Group A (*n* = 87)**	**Group B (*n* = 17)**	**Group C (*n* = 148)**	**Group D (*n* = 255)**	***P*-value**
Age (year)	71 ± 13	71 ± 12	70 ± 11	65 ± 13	<0.0001
Gender (male)	56 (64)	11 (65)	83 (56)	155 (61)	0.6024
Smoking	10 (11)	2 (12)	26 (18)	41 (16)	0.6182
**BP measurement**					0.1526
Home (*n* = 288) (56.8%)	54 (62)	9 (53)	73 (49)	152 (60)	
Office (*n* = 219) (43.2%)	33 (38)	8 (47)	75 (51)	103 (40)	
**Summertime BP**					
Systolic BP (mmHg)	126 ± 9	136 ± 15	133 ± 12	128 ± 9	<0.0001
Diastolic BP (mmHg)	72 ± 7	76 ± 7	75 ± 9	75 ± 8	0.0172
**Wintertime BP**					
Systolic BP (mmHg)	140 ± 10	124 ± 14	136 ± 12	129 ± 9	<0.0001
Diastolic BP (mmHg)	78 ± 8	73 ± 7	77 ± 9	76 ± 7	0.0541
**Chronic kidney disease**					0.0388
Stage 1	4 (5)	0	7 (5)	14 (5)	
Stage 2	2 (2)	2 (12)	10 (7)	27 (11)	
Stage 3	44 (51)	7 (41)	78 (53)	144 (56)	
Stage 4	30 (34)	5 (29)	49 (33)	57 (22)	
Stage 5	7 (8)	3 (18)	4 (3)	13 (5)	
Diabetic nephropathy	39 (45)	5 (29)	76 (51)	78 (31)	0.0003
Diabetes mellitus	39 (45)	7 (41)	84 (57)	95 (37)	0.0023
Hypertension	72 (83)	11 (65)	125 (84)	189 (74)	0.0321
Coronary artery disease	29 (33)	7 (41)	49 (33)	58 (23)	0.0449
Stroke	16 (18)	1 (6)	18 (12)	30 (12)	0.3261
Dyslipidemia	58 (67)	13 (76)	101 (68)	169 (66)	0.8384
Malignancy	20 (23)	2 (12)	35 (24)	60 (24)	0.7333
Heart failure	13 (15)	5 (29)	29 (20)	24 (9)	0.0084
Autoimmune disease	3 (3)	0	2 (1)	4 (2)	0.5894
Medication					
Nephrotoxic agents (NSAIDs, contrast media, aminoglycosides)	30 (34)	4 (24)	40 (27)	59 (23)	0.2182
ACEIs or ARBs	47 (54)	6 (35)	83 (56)	133 (52)	0.4214
β-blockers	12 (14)	3 (18)	24 (16)	44 (17)	0.8986
Calcium channel blockers	43 (49)	5 (29)	62 (42)	97 (38)	0.2151
α-blockers	12 (14)	1 (6)	8 (5)	21 (8)	0.1564
Diuretics	12 (14)	2 (12)	16 (11)	26 (10)	0.8319
Statins	28 (32)	4 (24)	66 (45)	134 (53)	0.0021
Antiplatelet agents	22 (25)	5 (29)	45 (30)	70 (27)	0.8502
BUN (mg/dL)	34 ± 14	38 ± 20	31 ± 14	30 ± 13	0.0137
Creatinine (mg/dL)	2.1 ± 1.0	2.3 ± 1.2	1.9 ± 0.8	1.8 ± 0.9	0.0329
eGFR (ml/min/1.73 m^2^)	36 ± 18	35 ± 20	39 ± 19	42 ± 21	0.0318
Albumin (g/dL)	4.3 ± 0.3	4.1 ± 0.4	4.2 ± 0.3	4.3 ± 0.4	0.1486
Hemoglobin (g/dL)	11.9 ± 1.8	12.0 ± 2.2	12.2 ± 1.8	12.6 ± 2.0	0.0076
Urine protein-creatinine ratio (PCR) (mg/g) mean ± SD	921 ± 981	1009 ± 1038	857 ± 1536	794 ± 1411	0.8326
median (interquartile range)	649	817	418	345	0.0185
	(210–1103)	(174–1590)	(192–880)	(134–911)	
Overall length of follow up (m)	54 ± 9	53 ± 11	56 ± 8	57 ± 7	0.0164
eGFR decline rate (ml/min/1.73 m^2^ per year) mean ± SD	3.0 ± 2.6	2.7 ± 3.0	2.8 ± 2.4	2.4 ± 2.6	0.1779
median (interquartile range)	2.6 (1.2–3.8)	2.3 (0–4.8)	2.6 (1.0–4.0)	2.0 (0.6–3.2)	0.0452
**Composite outcome**					
eGFR decline ≥40%, ESRD, or death	51 (59)	8 (47)	69 (47)	93 (36)	0.0031

*Data are presented as mean ± SD or number (percentage). Group A, BP elevation in the wintertime; Group B, BP reduction in the wintertime; Group C, large variation of BP; Group D, stable BP; CKD, chronic kidney disease; BP, blood pressure; NSAIDs, non-steroidal anti-inflammatory drugs; ACEIs, angiotensin converting enzyme inhibitors; ARBs, angiotensin receptor blockers; BUN, blood urea nitrogen; eGFR, estimated glomerular filtration rate; PCR, protein-creatinine ratio; ESRD, end stage renal disease*.

The comparison between included and excluded patients is shown in Supplementary Table 1. Compared to patients in the inclusion group, patients in the exclusion groups (group 1: dialysis, transplantation, or death; group 2: loss to follow-up) were older, had a higher prevalence in female gender, diabetes, hypertension and heart failure, and had a very high prevalence of advanced CKD (CKD stages 4 and 5) (70% in group 1 and 47% in group 2, respectively). The data indicated that patients in the exclusion groups may not represent a common CKD population.

Patients with BP elevation in the wintertime were older, and they had a higher prevalence of diabetic nephropathy and poorer outcome than the other groups. Patients with consistently lower BP in wintertime had a higher prevalence of coronary artery disease (CAD) and heart failure than the other groups. Compared with those in the other groups, patients with stable BP were younger and had a lower prevalence of diabetes mellitus, diabetic nephropathy, CAD, and heart failure, higher prevalence of statin use, higher values for estimated glomerular filtration rate (eGFR) and hemoglobin, and a more favorable outcome (Table 1).

Of the study population, 56.8% of patients conducted regular home BP monitoring. Their baseline characteristics were compared with those patients with only office BP measurement (Table 2). Patients with home BP monitoring had a higher prevalence of statin use (57 vs. 31%, *P* < 0.0001), higher hemoglobin levels (12.5 ± 1.9 g/dL vs. 12.1 ± 1.9 g/dL, *P* = 0.0436) and more favorable baseline renal function (eGFR 43 ± 21 ml/min/1.73 m^2^ vs. 35 ± 16 ml/min/1.73 m^2^, *P* < 0.0001) and composite outcome (39 vs. 49%, *P* = 0.0242). Table 3 presents the characteristics of the 288 patients with home BP monitoring in relation to the pattern of seasonal BP change. Compared with the other three groups, patients with BP elevation in the wintertime (Group A) were older, had a higher prevalence of diabetic nephropathy and nephrotoxic agent use, a lower prevalence of statin use, higher eGFR decline rate, and the worst outcome. Patients with BP reduction in the wintertime (Group B) were associated with the best outcome. Table 4 lists the characteristics of the 288 patients with CKD with available data of home BP monitoring in relation BP elevation in the wintertime. Patients with consistent BP elevation in wintertime were older (74 ± 9 vs. 67 ± 13 years, *P* = 0.0002) and had higher prevalence of diabetic nephropathy (54 vs. 37%, *P* = 0.0311), lower prevalence of statin use (41 vs. 61%, *P* = 0.0094), lower baseline eGFR (37 ± 19 ml/min/1.73 m^2^ vs. 45 ± 22 ml/min/1.73 m^2^, *P* = 0.0198), and worse outcome (57 vs. 35%, *P* = 0.0032) than those in the others.

**Table 2:** Characteristics of 507 CKD patients in relation to home BP and office BP measurements.

**Characteristic**	**Home BP monitoring (*n* = 288)**	**Office BP measurement (*n* = 219)**	***P*-value**
Age (year)	68 ± 12	68 ± 13	0.7530
Gender (male)	171 (59)	134 (61)	0.7145
Smoking	48 (17)	31 (14)	0.4608
Pattern of seasonal BP change			0.1526
Group A	54 (19)	33 (15)	
Group B	9 (3)	8 (4)	
Group C	73 (25)	75 (34)	
Group D	152 (53)	103 (47)	
**Summertime BP**			
Systolic BP (mmHg)	129 ± 10	130 ± 11	0.1409
Diastolic BP (mmHg)	74 ± 7	74 ± 9	0.6732
**Wintertime BP**			
Systolic BP (mmHg)	132 ± 11	134 ± 12	0.0464
Diastolic BP (mmHg)	76 ± 7	77 ± 8	0.5009
Chronic kidney disease			<0.0001
Stage 1	21 (7)	4 (2)	
Stage 2	31 (11)	10 (5)	
Stage 3	159 (55)	114 (52)	
Stage 4	61 (21)	80 (37)	
Stage 5	16 (6)	11 (5)	
Diabetic nephropathy	116 (40)	82 (37)	0.5218
Diabetes mellitus	132 (46)	93 (42)	0.4712
Hypertension	223 (77)	174 (79)	0.6637
Coronary artery disease	78 (27)	65 (30)	0.5506
Stroke	30 (10)	35 (16)	0.0807
Dyslipidemia	200 (69)	141 (64)	0.2519
Malignancy	65 (23)	52 (24)	0.8316
Heart failure	39 (14)	32 (15)	0.7964
Autoimmune disease	7 (2)	2 (1)	0.3114
**Medication**			
Nephrotoxic agents (NSAIDs, contrast media, aminoglycosides)	80 (28)	53 (24)	0.4150
ACEIs or ARBs	157 (55)	112 (51)	0.4731
β-blockers	44 (15)	39 (18)	0.4687
Calcium channel blockers	121 (42)	86 (39)	0.5843
α-blockers	30 (10)	12 (5)	0.0513
Diuretics	27 (9)	29 (13)	0.1982
Statins	164 (57)	68 (31)	<0.0001
Antiplatelet agents	91 (32)	51 (23)	0.0457
BUN (mg/dL)	29 ± 14	33 ± 14	0.0022
Creatinine (mg/dL)	1.8 ± 1.0	2.1 ± 1.9	0.0023
eGFR (ml/min/1.73 m^2^)	43 ± 21	35 ± 16	<0.0001
Albumin (g/dL)	4.3 ± 0.5	4.2 ± 0.4	0.2535
Hemoglobin (g/dL)	12.5 ± 1.9	12.1 ± 1.9	0.0436
Urine PCR (mg/g) mean ± SD	810 ± 1307	883 ± 1457	0.5506
Median (interquartile range)	445 (138–967)	413 (194–1028)	0.3984
Overall length of follow up (m)	56 ± 8	55 ± 8	0.3362
eGFR decline rate (ml/min/1.73 m^2^ per year) mean ± SD	2.6 ± 2.5	2.7 ± 2.6	0.7523
Median (interquartile range)	2.2 (0.6–3.7)	2.2 (0.8–3.8)	0.6970
**Composite outcome**			
eGFR decline ≥40%, ESRD, or death	113 (39)	108 (49)	0.0242

*Data are presented as mean ± SD or number (percentage). Group A, BP elevation in the wintertime; Group B, BP reduction in the wintertime; Group C, large variation of BP; Group D, stable BP; CKD, chronic kidney disease; BP, blood pressure; NSAIDs, non-steroidal anti-inflammatory drugs; ACEIs, angiotensin converting enzyme inhibitors; ARBs, angiotensin receptor blockers; BUN, blood urea nitrogen; eGFR, estimated glomerular filtration rate; PCR, protein-creatinine ratio; ESRD, end stage renal disease*.

**Table 3:** Characteristics of 288 CKD patients with home BP monitoring in relation to the pattern of seasonal BP change.

**Characteristic**	**Group A (*n* = 54)**	**Group B (*n* = 9)**	**Group C (*n* = 73)**	**Group D (*n* = 152)**	***P*-value**
Age (year)	74 ± 9	70 ± 12	70 ± 10	65 ± 14	<0.0001
Gender (male)	35 (65)	5 (56)	45 (62)	86 (57)	0.7137
Smoking	6 (11)	0	19 (26)	23 (15)	0.0489
**Summertime BP**					
Systolic BP (mmHg)	127 ± 9	132 ± 13	133 ± 12	127 ± 9	0.0006
Diastolic BP (mmHg)	72 ± 7	74 ± 6	75 ± 7	75 ± 8	0.0296
**Wintertime BP**					
Systolic BP (mmHg)	139 ± 10	120 ± 12	135 ± 12	129 ± 9	<0.0001
Diastolic BP (mmHg)	77 ± 8	71 ± 8	77 ± 7	76 ± 7	0.0735
Chronic kidney disease					0.0409
Stage 1	3 (6)	0	5 (7)	13 (9)	
Stage 2	1 (2)	1 (11)	7 (10)	22 (14)	
Stage 3	29 (54)	6 (67)	41 (56)	83 (55)	
Stage 4	17 (31)	0	19 (26)	25 (16)	
Stage 5	4 (7)	2 (22)	1 (1)	9 (6)	
Diabetic nephropathy	29 (54)	3 (33)	38 (52)	46 (30)	0.0020
Diabetes mellitus	29 (54)	3 (33)	43 (59)	57 (38)	0.0107
Hypertension	43 (80)	5 (56)	61 (84)	114 (75)	0.1953
Coronary artery disease	15 (28)	3 (33)	24 (33)	36 (24)	0.5085
Stroke	8 (15)	0	6 (8)	16 (11)	0.4671
Dyslipidemia	34 (63)	7 (78)	55 (75)	104 (68)	0.4513
Malignancy	11 (20)	0	18 (25)	36 (24)	0.3820
Heart failure	9 (17)	1 (11)	14 (19)	15 (10)	0.2378
Autoimmune disease	2 (4)	0	2 (3)	3 (2)	0.8598
**Medication**					
Nephrotoxic agents (NSAIDs, contrast media, aminoglycosides)	21 (39)	2 (22)	25 (34)	32 (21)	0.0382
ACEIs or ARBs	30 (56)	2 (22)	44 (60)	81 (53)	0.1810
β-blockers	6 (11)	2 (22)	12 (16)	24 (16)	0.7611
Calcium channel blockers	27 (50)	2 (22)	31 (42)	61 (40)	0.3783
α-blockers	8 (15)	0	6 (8)	16 (11)	0.4671
Diuretics	8 (15)	1 (11)	6 (8)	12 (8)	0.4900
Statins	22 (41)	2 (22)	43 (59)	97 (64)	0.0041
Antiplatelet agents	20 (37)	3 (33)	25 (34)	43 (28)	0.6240
BUN (mg/dL)	33 ± 14	31 ± 21	29 ± 12	28 ± 14	0.1652
Creatinine (mg/dL)	2.1 ± 1.1	2.2 ± 1.5	1.7 ± 0.8	1.7 ± 1.0	0.0959
eGFR (ml/min/1.73 m^2^)	37 ± 19	40 ± 23	42 ± 20	46 ± 22	0.0565
Albumin (g/dL)	4.3 ± 0.4	4.2 ± 0.4	4.2 ± 0.3	4.3 ± 0.4	0.7907
Hemoglobin (g/dL)	12.0 ± 1.9	12.5 ± 2.4	12.2 ± 1.8	12.8 ± 1.9	0.0652
Urine PCR (mg/g) mean ± SD	973 ± 1112	701 ± 610	789 ± 1064	768 ± 1498	0.7834
Median (interquartile range)	645	683	419	352	0.0937
	(146–1098)	(128–1031)	(171–879)	(130–886)	
Overall length of follow up (m)	54 ± 9	56 ± 11	56 ± 7	57 ± 7	0.0528
eGFR decline rate (ml/min/1.73 m^2^ per year) mean ± SD	3.1 ± 2.6	1.1 ± 1.8	3.0 ± 2.4	2.3 ± 2.5	0.0154
Median (interquartile range)	2.8 (1.2–3.9)	0.2 (0–2.4)	2.8 (1.2–4.1)	1.9 (0–3.0)	0.0017
Composite outcome	31 (57)	2 (22)	34 (47)	46 (30)	0.0015
eGFR decline ≥40%, ESRD, or death	–	–	–	–	–

*Data are presented as mean ± SD or number (percentage). Group A, BP elevation in the wintertime; Group B, BP reduction in the wintertime; Group C, large variation of BP; Group D, stable BP; CKD, chronic kidney disease; BP, blood pressure; NSAIDs, non-steroidal anti-inflammatory drugs; ACEIs, angiotensin converting enzyme inhibitors; ARBs, angiotensin receptor blockers; BUN, blood urea nitrogen; eGFR, estimated glomerular filtration rate; PCR, protein-creatinine ratio; ESRD, end stage renal disease*.

**Table 4:** Characteristics of 288 CKD patients with home BP monitoring in relation to BP elevation in the wintertime.

**Characteristic**	**BP elevation in the wintertime (+) (*n* = 54)**	**BP elevation in the wintertime (−) (*n* = 234)**	***P*-value**
Age (year)	74 ± 9	67 ± 13	0.0002
Gender (male)	35 (65)	136 (58)	0.4426
Smoking	6 (11)	42 (18)	0.3107
**Summertime BP**			
Systolic BP (mmHg)	127 ± 9	129 ± 11	0.1174
Diastolic BP (mmHg)	72 ± 7	75 ± 7	0.0035
**Wintertime BP**			
Systolic BP (mmHg)	139 ± 10	130 ± 11	<0.0001
Diastolic BP (mmHg)	77 ± 8	76 ± 7	0.1645
Chronic kidney disease			0.0625
Stage 1	3 (6)	18 (8)	
Stage 2	1 (2)	30 (13)	
Stage 3	29 (54)	130 (56)	
Stage 4	17 (31)	44 (19)	
Stage 5	4 (7)	12 (5)	
Diabetic nephropathy	29 (54)	87 (37)	0.0311
Diabetes mellitus	29 (54)	103 (44)	0.2265
Hypertension	43 (80)	180 (77)	0.7220
Coronary artery disease	15 (28)	63 (27)	0.8670
Stroke	8 (15)	22 (9)	0.2270
Dyslipidemia	34 (63)	166 (71)	0.2556
Malignancy	11 (20)	54 (23)	0.7220
Heart failure	9 (17)	30 (13)	0.5077
Autoimmune disease	2 (4)	5 (2)	0.6190
**Medication**			
Nephrotoxic agents (NSAIDs, contrast media, aminoglycosides)	21 (39)	59 (25)	0.0626
ACEIs or ARBs	30 (56)	127 (54)	0.8808
β-blockers	6 (11)	38 (16)	0.4075
Calcium channel blockers	27 (50)	94 (40)	0.2214
α-blockers	8 (15)	22 (9)	0.2270
Diuretics	8 (15)	19 (8)	0.1912
Statins	22 (41)	142 (61)	0.0094
Antiplatelet agents	20 (37)	71 (30)	0.3357
BUN (mg/dL)	33 ± 14	29 ± 14	0.0315
Creatinine (mg/dL)	2.1 ± 1.1	1.7 ± 0.9	0.0311
eGFR (ml/min/1.73 m^2^)	37 ± 19	45 ± 22	0.0198
Albumin (g/dL)	4.3 ± 0.4	4.3 ± 0.4	0.8914
Hemoglobin (g/dL)	12.0 ± 1.9	12.6 ± 1.9	0.0605
Urine PCR (mg/g) mean ± SD	973 ± 1111	772 ± 1348	0.3085
Median (interquartile range)	645 (146–1098)	389 (135–882)	0.0276
Overall length of follow up (m)	54 ± 9	57 ± 7	0.0105
eGFR decline rate (ml/min/1.73 m^2^ per year) mean ± SD	3.1 ± 2.6	2.4 ± 2.5	0.0897
Median (interquartile range)	2.8 (1.2–3.9)	2.0 (0.4–3.5)	0.0495
**Composite outcome**			
eGFR decline ≥40%, ESRD, or death	31 (57)	82 (35)	0.0032

*Data are presented as mean ± SD or number (percentage); Group A, BP elevation in the wintertime; Group B, BP reduction in the wintertime; Group C, large variation of BP; Group D, stable BP; CKD, chronic kidney disease; BP, blood pressure; NSAIDs, non-steroidal anti-inflammatory drugs; ACEIs, angiotensin converting enzyme inhibitors; ARBs, angiotensin receptor blockers; BUN, blood urea nitrogen; eGFR, estimated glomerular filtration rate; PCR, protein-creatinine ratio; ESRD, end stage renal disease*.

Because whether BP elevation in the wintertime demonstrated violation of the proportional hazards' assumption, a stratified Cox regression analysis on the stratum of BP elevation in the wintertime was performed. It showed home BP monitoring was independently associated with a better outcome in 507 CKD patients (HR 0.72, 95% CI 0.56–0.94, *P* = 0.0162) (Table 5). Cox regression analysis for association between clinical factors and poor outcome (eGFR decline ≥40%, dialysis, transplantation, or death) in CKD patients with home BP measurement is shown in Table 6. Patients with CKD who conducted home BP measurement and experienced poor outcomes were more likely to have BP elevation in wintertime and heart failure, had lower levels of baseline eGFR, serum albumin and hemoglobin, and had higher levels of blood urea nitrogen, serum creatinine, urine protein-creatinine ratio (PCR), and eGFR decline rate. Multivariable Cox regression analysis revealed that a consistent BP elevation in the wintertime in CKD patients with home BP monitoring was significantly associated with a worse outcome after adjustment for age, gender and smoking as well as adjustment for age, gender, smoking, heart failure, diabetic nephropathy, eGFR, albumin, hemoglobin, urine PCR, and ACEIs/ARBs (HR: 2.09, 95% CI: 1.37–3.20, *P* = 0.0007; HR: 1.72, 95% CI: 1.09–2.72, *P* = 0.0205, respectively) (Table 7).

**Table 5:** Cox regression analysis (on the stratum of BP elevation in the wintertime) for factors associated with poor outcome (eGFR decline ≥40%, dialysis, transplantation, or death) in 507 CKD patients.

**Variable**	**HR**	**95% CI**	***P*-value**
Home BP monitoring	0.72	0.56–0.94	0.0162
Age (year)	1.00	0.99–1.01	0.8555
Gender (male)	0.84	0.64–1.11	0.2233
Smoking	0.72	0.47–1.11	0.1334

*CKD, chronic kidney disease; BP, blood pressure*.

**Table 6:** Cox regression analysis for association between clinical factors and poor outcome (eGFR decline ≥40%, dialysis, transplantation, or death) in 288 CKD patients with home BP monitoring.

**Variable**	**HR (95% CI)**	***P*-value**
Age (year)	1.01 (0.99–1.02)	0.3547
Gender (male)	0.73 (0.50–1.05)	0.0893
Smoking	0.84 (0.50–1.40)	0.5023
BP elevation in the wintertime	2.09 (1.39–3.15)	0.0004
Diabetic nephropathy	1.20 (0.83–1.74)	0.3303
Diabetes mellitus	1.22 (0.84–1.76)	0.2979
Hypertension	1.60 (0.98–2.62)	0.0605
Coronary artery disease	1.07 (0.71–1.61)	0.7528
Stroke	1.13 (0.65–1.98)	0.6713
Dyslipidemia	0.85 (0.58–1.26)	0.4232
Malignancy	1.04 (0.68–1.60)	0.8431
Heart failure	1.78 (1.13–2.81)	0.0138
Autoimmune disease	1.04 (0.33–3.28)	0.9466
Nephrotoxic agents (NSAIDs, contrast media, aminoglycosides)	1.27 (0.86–1.89)	0.2296
ACEIs or ARBs	0.89 (0.61–1.29)	0.5223
β-blockers	0.67 (0.39–1.18)	0.1664
Calcium channel blockers	1.19 (0.82–1.72)	0.3560
α-blockers	1.25 (0.71–2.18)	0.4432
Diuretics	2.33 (1.37–3.96)	0.0018
Statins	0.86 (0.59–1.24)	0.4059
Antiplatelet agents	1.14 (0.78–1.68)	0.5011
BUN (mg/dL)	1.04 (1.03–1.05)	<0.0001
Creatinine (mg/dL)	1.74 (1.50–2.00)	<0.0001
eGFR (ml/min/1.73 m^2^)	0.97 (0.96–0.98)	<0.0001
Albumin (g/dL)	0.65 (0.43–0.97)	0.0352
Hemoglobin (g/dL)	0.74 (0.67–0.83)	<0.0001
Urine PCR (mg/g)	1.00 (1.00–1.01)	0.0007
eGFR decline rate (ml/min/1.73 m^2^ per year)	1.26 (1.19–1.32)	<0.0001

*CKD, chronic kidney disease; BP, blood pressure; eGFR, estimated glomerular filtration rate; NSAIDs, non-steroidal anti-inflammatory drugs; ACEIs, angiotensin converting enzyme inhibitors; ARBs, angiotensin receptor blockers; BUN, blood urea nitrogen; PCR, protein-creatinine ratio; ESRD, end stage renal disease*.

**Table 7:** Hazard ratios for relationship between BP elevation in the wintertime and poor outcome (eGFR decline ≥40%, dialysis, transplantation, or death) in 288 CKD patients with home BP monitoring.

	**BP elevation in the wintertime**
	**HR (95% CI)**	***P*****-value**
Unadjusted	2.09 (1.39–3.15)	0.0004
Model 1	2.09 (1.37–3.20)	0.0007
Model 2	2.00 (1.31–3.07)	0.0015
Model 3	1.72 (1.09–2.72)	0.0205

*Model 1: adjusted for age, gender and smoking*.

*Model 2: adjusted for age, gender, smoking, diabetic nephropathy, and heart failure*.

*Model 3: adjusted for age, gender, smoking, heart failure, diabetic nephropathy, eGFR, albumin, hemoglobin, urine PCR, ACEIs or ARBs*.

*BP, blood pressure; eGFR, estimated glomerular filtration rate; PCR, protein-creatinine ratio; ACEIs, angiotensin converting enzyme inhibitors; ARBs, angiotensin receptor blockers*.

## Discussion

Our review of the literature suggests that this study is the first to demonstrate the association between seasonal BP change and outcome in patients with CKD. Using real-world data related to the management of patient with CKD, we endeavored to highlight the importance of home BP monitoring. According to the pattern of seasonal BP change based on home BP measurement, a consistently elevated BP in the wintertime in those with CKD was associated with a worse composite patient outcome (i.e., ≥40% reduction in eGFR, dialysis, transplantation, or death).

Hanazawa et al. reported that patients with large BP variation, defined as an elevation of systolic BP ≥ 9.1 mmHg during winter, comprised 22.9% of their study population (6). By contrast, our study had a low percentage (17.2%) of patients with BP elevation in the wintertime. This disparity may be related to the modest temperature variation from summer to winter in southern Taiwan where our patients lived. According to data from the Central Weather Bureau of Taiwan on the mean ambient temperature in southern Taiwan over the past 30 years, during the warm months (May to October) the temperature is 27.5–28.6 °C, but it drops to 17.8–19.6 °C in the cooler months (December to February). Moreover, patients were categorized as having consistent BP elevation in wintertime only if they had systolic BP ≥ 8 mmHg throughout the study period. Because this was an observational study, no standardized BP measurement protocol was employed; thus, the BP differences in numerous measurements must be sufficiently large to enable the study to attain reliable results. According to a study by Hanazawa et al., cardiovascular risk increased significantly only in patients with a larger seasonal variation in home BP (i.e., >9.1/4.5 mmHg) (6).

Related studies have generally reported that home BP monitoring has a higher predictive value than office BP measurements. A study on the prognostic significance of home BP control on renal and cardiovascular outcomes in older patients with CKD revealed the importance of home BP in predicting renal function deterioration (14). Celis et al. reported a 17.2% elevation in cardiovascular risk with each 10 mmHg increase of systolic home BP and 11.7% with each 5 mmHg increase of diastolic home BP. However, a similar increase in the office BP was not associated with a significant increase in cardiovascular risk (15). Our study revealed that the significant differences observed in the clinical characteristics of the 507 patients with CKD (with and without home BP monitoring) between different patterns of seasonal BP change disappeared when further analysis focused on the 288 patients with CKD with home BP monitoring. When only the data of patients with home BP monitoring were used in the analysis, in comparison with the others, patients with consistent BP elevation in the wintertime had significantly lower eGFR and higher urine UPCR, and exhibited a significantly higher eGFR decline rate and worse outcome. Although the notion of home BP monitoring has been addressed in the literature and highlighted for more than a decade, only 56.8% of our patients performed regular home BP measurement. Clearly, the attitude and awareness of physicians and patients toward home BP measurement affect patient adherence and outcomes (16, 17).

Our study revealed that patients with CKD with an elevated home-measured BP in the wintertime were significantly older and were more likely to have diabetic nephropathy and advanced CKD stages. Age-related endothelial dysfunction is inevitable with advancing age (17, 18). It is related to a reduction in endothelial nitric oxide synthase (eNOS) and increased oxygen radicals in the aging process (17). Hyperglycemia affects the bioavailability of nitric oxide and increases the number of oxygen radicals and endothelium-derived vasoconstrictors such as endothelin-1, prostanoids, and angiotensin II (16). Accumulation of asymmetric dimethylarginine, a potent endogenous inhibitor of eNOS in patients with advanced CKD, results in endothelial dysfunction (19). The autoregulatory ability in response to RAS and sympathetic activation during the cold seasons is evidently impaired in these patients. The outcomes of patients with CKD with consistently elevated BP in the wintertime were clearly inferior to the outcomes of those with large BP variation but no apparent seasonal patterns, BP reduction in wintertime, and stable BP. The exact mechanism for this phenomenon remains unclear. The mechanism of BP elevation in cold weather and the subsequent poorer renal and patient outcomes may be partially explained by RAS and sympathetic overactivation, endothelial dysfunction, and vitamin D deficiency (20–23).

The exact mechanisms for the association of other BP groups with renal outcomes in CKD patients are not clear. The visit-visit BP variability has been proved to be a good predictor of cardiovascular risk and all-cause mortality, especially in hypertensive patients on treatment (24). The underlying cardiovascular risk level has impact on the association between visit-visit BP variability and cardiovascular risk, and visit-visit BP variability could be a strong predictor of cardiovascular events and mortality in high-risk population (25). The major determinants for long-term BP variability (visit to visit) are improper dosage/titration of antihypertensive therapy, increased arterial stiffness, aging, reduced adherence to therapy, and seasonal changes (25, 26). Inverse seasonal variation in BP (higher BP in summer) can be observed mainly in patients treated with antihypertensive agents, possibly reflecting an abnormal response of biological pathways to the outside temperature. Inverse seasonal variation in BP may also related to lack of medication adherence and hard labor only during the summer. However, factors related to inverse seasonal variation need to be further investigated (6).

This study has several limitations. We analyzed the data of only 507 patients with CKD from a single tertiary center. This study's relatively small sample size, accompanied by few events, impeded the analysis of each component of the composite outcome because of limited statistical power. The BP and clinical data were collected from electronic medical records; thus, the consistency and accuracy of BP measurement could not be confirmed. Large interindividual and intraindividual BP variability helped reduce the specificity of the BP variation pattern; however, the impact of high BP on the seasonal BP change and outcomes was not evaluated in this study. Therefore, we emphasized the association between patient outcomes and BP change from summertime to wintertime instead of employing absolute BP value or BP control. Because this was a retrospective, cross sectional study, the results suggest an association between the clinical variables rather than a cause–effect relationship. Nevertheless, incorporating our findings-that effective BP control in the wintertime helps improve outcomes-into daily clinical practice is reasonable. A prospective, multicenter study with a larger sample size and longer follow-up duration is required to confirm that BP elevation in the wintertime is a major predictor of poor outcomes in patients with CKD.

In conclusion, we demonstrated the impact of seasonal BP change on the outcomes of patients with CKD: those presenting consistent BP elevation from summertime to wintertime exhibited significantly worse outcomes. This phenomenon was observed in southern Taiwan, where has a modest temperature variation, in CKD patients with home BP monitoring.

## Data Availability Statement

The original contributions presented in the study are included in the article/Supplementary Material, further inquiries can be directed to the corresponding authors.

## Ethics Statement

This study involving human participants was reviewed and approved by the Institutional Review Board of National Cheng Kung University Hospital. PS: Written informed consent was not required to this retrospective study.

## Author Contributions

CYS and M-CW contributed to the conception and design of the study. CYS organized the database and wrote the first draft of the manuscript. M-CW performed the statistical analysis. M-CW and T-HK revising it critically for important intellectual content. All authors contributed to the article and approved the submitted version.

## Conflict of Interest

The authors declare that the research was conducted in the absence of any commercial or financial relationships that could be construed as a potential conflict of interest.
