# Supplementary material for: Seasonal Change in Home Blood Pressure Monitoring Is Associated With Renal Outcome and Mortality in Patients With Chronic Kidney Disease
Source: Front Med (Lausanne). 2021 May 28;8:672651. doi: 10.3389/fmed.2021.672651 (PMC8192714; doi:10.3389/fmed.2021.672651)
Supplement: Supplementary file 1 [file Table_1.docx]

**Supplementary Material**

**Supplementary Table 1.** Comparison of baseline characteristics between the inclusion and exclusion groups

| Characteristic | Inclusion group  (n = 507) | Exclusion group 1  (n = 226) | Exclusion group 2  (n = 650) | P value |
| --- | --- | --- | --- | --- |
| Age (year) | 68 ± 13 | 77 + 14 | 72 ± 14 | <0.0001 |
| Gender (male) | 305 (60) | 85 (38) | 263 (40) | <0.0001 |
| BUN (mg/L) | 31 ± 14 | 50 ± 26 | 37 ± 18 | <0.0001 |
| Creatinine (mg/dL) | 1.9 ± 0.9 | 5.4 ± 10.9 | 3.0 ± 5.6 | <0.0001 |
| eGFR (ml/min/1.73 m^2^) | 39.8 ± 19.7 | 22.7 ± 15.0 | 31.4 ± 16.9 | <0.0001 |
| Chronic kidney disease |  |  |  | <0.0001 |
| Stage 1 | 25 (5) | 2 (1) | 17 (3) |  |
| Stage 2 | 41 (8) | 4 (2) | 45 (7) |  |
| Stage 3 | 273 (54) | 62 (27) | 284 (44) |  |
| Stage 4 | 141 (28) | 72 (32) | 189 (29) |  |
| Stage 5 | 27 (5) | 86 (38) | 115 (18) |  |
| Diabetes mellitus | 225 (44) | 136 (60) | 332 (51) | 0.0003 |
| Hypertension | 397 (78) | 204 (90) | 574 (88) | <0.0001 |
| Coronary artery disease | 143 (28) | 63 (28) | 142 (22) | 0.0277 |
| Heart failure | 71 (14) | 50 (22) | 95 (15) | 0.0125 |
| Albumin (g/dL) | 4.3 ± 0.4 | 4.0 ± 0.5 | 4.2 ± 0.4 | <0.0001 |
| Hemoglobin (g/dL) | 12.3 ± 1.9 | 10.7 ± 1.9 | 11.8 ± 2.0 | <0.0001 |
| Urine PCR (mg/g) | 841 ± 1373 | 2196 ± 2597 | 1315 ± 1760 | <0.0001 |

Data are presented as mean ± SD or number (percentage)

Exclusion group 1: dialysis, transplantation, or death

Exclusion group 2: loss to follow-up
